# (*E*)-1-(2,5-Dichloro-3-thien­yl)-3-(3,4-dimeth­oxy­phen­yl)prop-2-en-1-one

**DOI:** 10.1107/S1600536810035142

**Published:** 2010-09-04

**Authors:** William T. A. Harrison, C. S. Chidan Kumar, H. S. Yathirajan, A. N. Mayekar, B. Narayana

**Affiliations:** a Department of Chemistry, University of Aberdeen, Meston Walk, Aberdeen AB24 3UE, Scotland; b Department of Studies in Chemistry, University of Mysore, Manasagangotri, Mysore 570 006, India; c SeQuent Scientific Limited, New Mangalore 575 011, India; d Department of Chemistry, Mangalore University, Mangalagangotri 574 199, India

## Abstract

In the title compound, C_15_H_12_Cl_2_O_3_S, the prop-2-en-1-one (enone) fragment is almost planar [C—C—C—O = 2.2 (4)°] and it subtends dihedral angles of 11.9 (2) and 11.0 (2)° with the thio­phene and benzene rings, respectively. The dihedral angle between the aromatic rings is 3.47 (16)°. In the crystal, weak C—H⋯O and C—H⋯Cl inter­actions link the mol­ecules, leading to *R*_2_^2^(14), *R*_2_^2^(24) and *C*(11) supra­molecular motifs occurring within the three-dimensional network. Weak aromatic π–π stacking [centroid–centroid separations = 3.6823 (15) and 3.8722 (15) Å] may also help to consolidate the packing.

## Related literature

For a related structure and background references, see: Jasinski *et al.* (2010 ▶). For reference structural data, see: Allen *et al.* (1987 ▶).
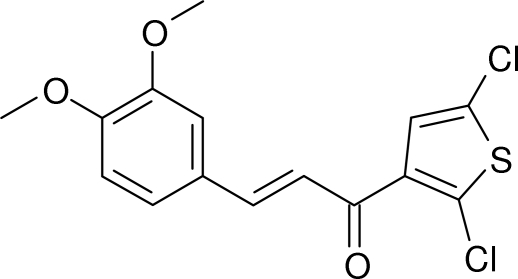


## Experimental

### 

#### Crystal data

C_15_H_12_Cl_2_O_3_S
*M* *_r_* = 343.21
Monoclinic, 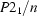

*a* = 8.9331 (2) Å
*b* = 8.9997 (2) Å
*c* = 18.8210 (5) Å
β = 100.181 (1)°
*V* = 1489.29 (6) Å^3^
*Z* = 4
Mo *K*α radiation
μ = 0.58 mm^−1^
*T* = 120 K
0.24 × 0.12 × 0.10 mm

#### Data collection

Nonius KappaCCD diffractometer
Absorption correction: multi-scan [*SADABS* (Bruker, 2003 ▶) and Blessing (1995 ▶)] *T*_min_ = 0.873, *T*_max_ = 0.944
22032 measured reflections
3424 independent reflections
2834 reflections with *I* > 2σ(*I*)
*R*_int_ = 0.056

#### Refinement

*R*[*F* ^2^ > 2σ(*F* ^2^)] = 0.048
*wR*(*F* ^2^) = 0.113
*S* = 1.10
3424 reflections
193 parameters
H-atom parameters constrained
Δρ_max_ = 0.72 e Å^−3^
Δρ_min_ = −0.41 e Å^−3^

### 

Data collection: *COLLECT* (Nonius, 1998 ▶); cell refinement: *COLLECT*; data reduction: *DENZO* (Otwinowski & Minor, 1997 ▶); program(s) used to solve structure: *SHELXS97* (Sheldrick, 2008 ▶); program(s) used to refine structure: *SHELXL97* (Sheldrick, 2008 ▶); molecular graphics: *ORTEP-3* (Farrugia, 1997 ▶); software used to prepare material for publication: *publCIF* (Westrip, 2010 ▶).

## Supplementary Material

Crystal structure: contains datablocks I, global. DOI: 10.1107/S1600536810035142/ng5014sup1.cif

Structure factors: contains datablocks I. DOI: 10.1107/S1600536810035142/ng5014Isup2.hkl

Additional supplementary materials:  crystallographic information; 3D view; checkCIF report

## Figures and Tables

**Table 1:** Hydrogen-bond geometry (Å, °)

*D*—H⋯*A*	*D*—H	H⋯*A*	*D*⋯*A*	*D*—H⋯*A*
C3—H3⋯O3^i^	0.95	2.53	3.227 (3)	130
C12—H12⋯O1^ii^	0.95	2.55	3.441 (3)	157
C14—H14*A*⋯O3^iii^	0.98	2.53	3.474 (3)	161
C15—H15*B*⋯Cl1^iv^	0.98	2.82	3.647 (3)	142

Symmetry codes: (i) 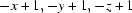; (ii) 
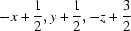
; (iii) 
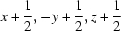
; (iv) 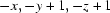.

**Fig. 1.**
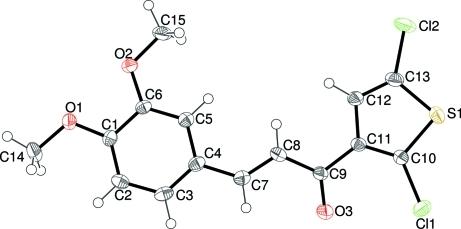
View of the molecular structure of (I) showing 50% displacement ellipsoids (arbitrary spheres for the H atoms).

## supplementary crystallographic information

### Comment

The title compound, (I), (Fig. 1), was prepared as part of our ongoing structural studies (Jasinski *et al.*, 2010) of chalcone-like compounds, in which substituted aromatic ring systems are linked by a prop-2-en-1-one bridge.

The prop-2-en-1-one fragment in (I) is almost planar [C7—C8—C9—O3 = 2.2 (4)°] and it subtends dihedral angles of 11.9 (2) and 11.0 (2)° with the thiophene and benzene rings, respectively. The dihedral angle between the aromatic rings is 3.47 (16)°. The carbon atoms of the methoxy groups are close to co-planar with their attached benzene ring [displacements of 0.033 (5) and 0.100 (5)Å for C14 and C15, respectively]. Otherwise, the bond lengths for (I) fall within their expected ranges (Allen *et al.*, 1987) and are similar to those in a related structure (Jasinski *et al.*, 2010).

In the crystal, three weak C—H···O and one C—H···Cl interactions (Table 1) link the molecules. Considered individually, they generate the following motifs: the C3—H3 bond generates inversion dimers containing *R*_2_^2^(14) rings, whereas the C12—H12 bond leads to C(11) chains propagating in [010]. The methyl-H bonds lead to inversion-generated *R*_2_^2^(24) loops (for C15—H15B) and C(11) chains (for C14—H14A). Taken together, these four interactions generate a three-dimensional network. Weak aromatic π-π stacking [centroid-centroid separations = 3.6823 (15) and 3.8722 (15) Å] may also help to consolidate the packing.

### Experimental

2,5-Dichloro-3-acetylthiophene was obtained as a gift sample from SeQuent Scientific ltd., New Mangalore, India. 1-(2,5-Dichlorothiophen-3-yl)ethanone (1.95 g, 0.01 mol) was mixed with 3,4-dimethoxybenzaldehyde (1.66 g, 0.01 mol) and dissolved in ethanol (30 ml). To this, 3 ml of 50% KOH was added. The reaction mixture was stirred for 6 h. The resulting crude solid was filtered, washed successively with distilled water and finally recrystallized from ethanol (95%) to give the pure chalcone. Irregular yellow crystals of (I) were obtained by the slow evaporation of DMF solution (m.p.: 389–391 K).

### Refinement

The hydrogen atoms were geometrically placed (C—H = 0.95–0.98 Å) and refined as riding with *U*_iso_(H) = 1.2*U*_eq_(C) or 1.5*U*_eq_(methyl C). A rotating group model was applied to the methyl group.

### Crystal data

**Table:**

C_15_H_12_Cl_2_O_3_S	*F*(000) = 704
*M**_r_* = 343.21	*D*_x_ = 1.531 Mg m^−^^3^
Monoclinic, *P*2_1_/*n*	Mo *K*α radiation, λ = 0.71073 Å
Hall symbol: -P 2yn	Cell parameters from 20728 reflections
*a* = 8.9331 (2) Å	θ = 2.9–27.5°
*b* = 8.9997 (2) Å	µ = 0.58 mm^−^^1^
*c* = 18.8210 (5) Å	*T* = 120 K
β = 100.181 (1)°	Fragment, yellow
*V* = 1489.29 (6) Å^3^	0.24 × 0.12 × 0.10 mm
*Z* = 4	

### Data collection

**Table:**

Nonius KappaCCD diffractometer	3424 independent reflections
Radiation source: fine-focus sealed tube	2834 reflections with *I* > 2σ(*I*)
graphite	*R*_int_ = 0.056
ω and φ scans	θ_max_ = 27.6°, θ_min_ = 3.2°
Absorption correction: multi-scan [*SADABS* (Bruker, 2003) and Blessing (1995)]	*h* = −11→11
*T*_min_ = 0.873, *T*_max_ = 0.944	*k* = −11→11
22032 measured reflections	*l* = −24→24

### Refinement

**Table:**

Refinement on *F*^2^	Secondary atom site location: difference Fourier map
Least-squares matrix: full	Hydrogen site location: inferred from neighbouring sites
*R*[*F*^2^ > 2σ(*F*^2^)] = 0.048	H-atom parameters constrained
*wR*(*F*^2^) = 0.113	*w* = 1/[σ^2^(*F*_o_^2^) + (0.034*P*)^2^ + 1.9239*P*] where *P* = (*F*_o_^2^ + 2*F*_c_^2^)/3
*S* = 1.10	(Δ/σ)_max_ < 0.001
3424 reflections	Δρ_max_ = 0.72 e Å^−^^3^
193 parameters	Δρ_min_ = −0.41 e Å^−^^3^
0 restraints	Extinction correction: *SHELXL97* (Sheldrick, 2008), Fc^*^=kFc[1+0.001xFc^2^λ^3^/sin(2θ)]^-1/4^
Primary atom site location: structure-invariant direct methods	Extinction coefficient: 0.011 (2)

### Special details

**Table:**

Geometry. All e.s.d.'s (except the e.s.d. in the dihedral angle between two l.s. planes) are estimated using the full covariance matrix. The cell e.s.d.'s are taken into account individually in the estimation of e.s.d.'s in distances, angles and torsion angles; correlations between e.s.d.'s in cell parameters are only used when they are defined by crystal symmetry. An approximate (isotropic) treatment of cell e.s.d.'s is used for estimating e.s.d.'s involving l.s. planes.
Refinement. Refinement of *F*^2^ against ALL reflections. The weighted *R*-factor *wR* and goodness of fit *S* are based on *F*^2^, conventional *R*-factors *R* are based on *F*, with *F* set to zero for negative *F*^2^. The threshold expression of *F*^2^ > σ(*F*^2^) is used only for calculating *R*-factors(gt) *etc*. and is not relevant to the choice of reflections for refinement. *R*-factors based on *F*^2^ are statistically about twice as large as those based on *F*, and *R*- factors based on ALL data will be even larger.

### Fractional atomic coordinates and isotropic or equivalent isotropic displacement parameters (Å^2^)

**Table:**

	*x*	*y*	*z*	*U*_iso_*/*U*_eq_	
C1	0.4943 (3)	0.2226 (3)	0.72975 (12)	0.0218 (5)	
C2	0.5681 (3)	0.2506 (3)	0.67246 (14)	0.0253 (5)	
H2	0.6632	0.2050	0.6705	0.030*	
C3	0.5013 (3)	0.3471 (3)	0.61723 (14)	0.0273 (5)	
H3	0.5512	0.3644	0.5774	0.033*	
C4	0.3644 (3)	0.4175 (3)	0.61939 (13)	0.0248 (5)	
C5	0.2908 (3)	0.3901 (3)	0.67817 (13)	0.0227 (5)	
H5	0.1977	0.4388	0.6809	0.027*	
C6	0.3533 (3)	0.2927 (3)	0.73192 (12)	0.0207 (5)	
C7	0.2948 (3)	0.5123 (3)	0.55890 (14)	0.0269 (5)	
H7	0.3459	0.5142	0.5187	0.032*	
C8	0.1686 (3)	0.5971 (3)	0.55235 (13)	0.0232 (5)	
H8	0.1154	0.6027	0.5918	0.028*	
C9	0.1115 (3)	0.6799 (3)	0.48719 (13)	0.0230 (5)	
C10	−0.0865 (3)	0.8706 (3)	0.43372 (13)	0.0217 (5)	
C11	−0.0291 (3)	0.7698 (3)	0.48589 (12)	0.0213 (5)	
C12	−0.1241 (3)	0.7591 (3)	0.53941 (13)	0.0265 (5)	
H12	−0.1035	0.6955	0.5803	0.032*	
C13	−0.2457 (3)	0.8490 (3)	0.52538 (14)	0.0298 (6)	
C14	0.6852 (3)	0.0533 (3)	0.78546 (16)	0.0382 (7)	
H14A	0.7073	−0.0128	0.8274	0.057*	
H14B	0.7672	0.1264	0.7875	0.057*	
H14C	0.6776	−0.0053	0.7411	0.057*	
C15	0.1441 (3)	0.3186 (3)	0.79360 (15)	0.0317 (6)	
H15A	0.1087	0.2819	0.8368	0.047*	
H15B	0.0709	0.2905	0.7505	0.047*	
H15C	0.1532	0.4270	0.7960	0.047*	
O1	0.5440 (2)	0.1289 (2)	0.78610 (9)	0.0284 (4)	
O2	0.28886 (19)	0.25494 (19)	0.78990 (9)	0.0265 (4)	
O3	0.1727 (2)	0.6738 (2)	0.43354 (10)	0.0417 (5)	
S1	−0.25196 (7)	0.95280 (7)	0.44816 (4)	0.02811 (19)	
Cl1	−0.01562 (8)	0.92675 (7)	0.35926 (3)	0.03212 (19)	
Cl2	−0.38786 (8)	0.86816 (11)	0.57543 (4)	0.0513 (3)	

### Atomic displacement parameters (Å^2^)

**Table:**

	*U*^11^	*U*^22^	*U*^33^	*U*^12^	*U*^13^	*U*^23^
C1	0.0237 (11)	0.0212 (12)	0.0189 (11)	−0.0020 (9)	−0.0004 (9)	−0.0012 (9)
C2	0.0203 (11)	0.0268 (13)	0.0298 (13)	−0.0032 (10)	0.0076 (10)	−0.0045 (10)
C3	0.0309 (13)	0.0281 (13)	0.0250 (13)	−0.0083 (11)	0.0105 (10)	0.0000 (10)
C4	0.0299 (13)	0.0216 (12)	0.0238 (12)	−0.0040 (10)	0.0073 (10)	0.0007 (10)
C5	0.0266 (12)	0.0204 (12)	0.0213 (12)	−0.0007 (9)	0.0050 (10)	−0.0023 (9)
C6	0.0256 (11)	0.0190 (11)	0.0182 (11)	−0.0033 (9)	0.0056 (9)	−0.0021 (9)
C7	0.0264 (12)	0.0298 (14)	0.0260 (13)	−0.0011 (10)	0.0084 (10)	0.0025 (10)
C8	0.0204 (11)	0.0237 (12)	0.0267 (12)	0.0006 (9)	0.0074 (10)	−0.0039 (10)
C9	0.0220 (11)	0.0271 (13)	0.0208 (12)	0.0034 (10)	0.0062 (9)	−0.0009 (10)
C10	0.0195 (11)	0.0242 (12)	0.0211 (12)	0.0005 (9)	0.0025 (9)	−0.0032 (9)
C11	0.0213 (11)	0.0247 (12)	0.0180 (11)	0.0003 (9)	0.0040 (9)	−0.0030 (9)
C12	0.0261 (12)	0.0357 (14)	0.0181 (12)	0.0048 (11)	0.0051 (10)	−0.0003 (10)
C13	0.0265 (12)	0.0410 (15)	0.0232 (13)	0.0034 (11)	0.0077 (10)	−0.0050 (11)
C14	0.0296 (14)	0.0439 (17)	0.0400 (16)	0.0158 (12)	0.0033 (12)	0.0039 (13)
C15	0.0320 (13)	0.0358 (15)	0.0307 (14)	0.0038 (11)	0.0152 (11)	−0.0037 (11)
O1	0.0297 (9)	0.0313 (10)	0.0242 (9)	0.0095 (8)	0.0045 (7)	0.0036 (7)
O2	0.0308 (9)	0.0277 (9)	0.0230 (9)	0.0043 (7)	0.0106 (7)	0.0036 (7)
O3	0.0426 (11)	0.0582 (14)	0.0277 (10)	0.0238 (10)	0.0159 (9)	0.0103 (9)
S1	0.0233 (3)	0.0303 (4)	0.0296 (3)	0.0064 (3)	0.0016 (2)	−0.0019 (3)
Cl1	0.0378 (4)	0.0324 (4)	0.0278 (3)	−0.0019 (3)	0.0102 (3)	0.0059 (3)
Cl2	0.0363 (4)	0.0820 (6)	0.0411 (4)	0.0219 (4)	0.0215 (3)	0.0036 (4)

### Geometric parameters (Å, °)

**Table:**

C1—O1	1.367 (3)	C9—C11	1.491 (3)
C1—C2	1.383 (3)	C10—C11	1.368 (3)
C1—C6	1.415 (3)	C10—Cl1	1.713 (2)
C2—C3	1.404 (4)	C10—S1	1.717 (2)
C2—H2	0.9500	C11—C12	1.430 (3)
C3—C4	1.385 (4)	C12—C13	1.343 (4)
C3—H3	0.9500	C12—H12	0.9500
C4—C5	1.405 (3)	C13—Cl2	1.718 (3)
C4—C7	1.470 (3)	C13—S1	1.720 (3)
C5—C6	1.380 (3)	C14—O1	1.435 (3)
C5—H5	0.9500	C14—H14A	0.9800
C6—O2	1.364 (3)	C14—H14B	0.9800
C7—C8	1.348 (3)	C14—H14C	0.9800
C7—H7	0.9500	C15—O2	1.427 (3)
C8—C9	1.448 (3)	C15—H15A	0.9800
C8—H8	0.9500	C15—H15B	0.9800
C9—O3	1.231 (3)	C15—H15C	0.9800
			
O1—C1—C2	125.7 (2)	C11—C10—Cl1	129.58 (18)
O1—C1—C6	114.9 (2)	C11—C10—S1	113.31 (18)
C2—C1—C6	119.4 (2)	Cl1—C10—S1	117.10 (14)
C1—C2—C3	119.4 (2)	C10—C11—C12	110.9 (2)
C1—C2—H2	120.3	C10—C11—C9	125.4 (2)
C3—C2—H2	120.3	C12—C11—C9	123.7 (2)
C4—C3—C2	121.5 (2)	C13—C12—C11	112.4 (2)
C4—C3—H3	119.2	C13—C12—H12	123.8
C2—C3—H3	119.2	C11—C12—H12	123.8
C3—C4—C5	118.8 (2)	C12—C13—Cl2	127.1 (2)
C3—C4—C7	119.9 (2)	C12—C13—S1	113.43 (19)
C5—C4—C7	121.3 (2)	Cl2—C13—S1	119.47 (16)
C6—C5—C4	120.3 (2)	O1—C14—H14A	109.5
C6—C5—H5	119.9	O1—C14—H14B	109.5
C4—C5—H5	119.9	H14A—C14—H14B	109.5
O2—C6—C5	124.9 (2)	O1—C14—H14C	109.5
O2—C6—C1	114.6 (2)	H14A—C14—H14C	109.5
C5—C6—C1	120.6 (2)	H14B—C14—H14C	109.5
C8—C7—C4	129.2 (2)	O2—C15—H15A	109.5
C8—C7—H7	115.4	O2—C15—H15B	109.5
C4—C7—H7	115.4	H15A—C15—H15B	109.5
C7—C8—C9	122.1 (2)	O2—C15—H15C	109.5
C7—C8—H8	118.9	H15A—C15—H15C	109.5
C9—C8—H8	118.9	H15B—C15—H15C	109.5
O3—C9—C8	122.1 (2)	C1—O1—C14	116.9 (2)
O3—C9—C11	120.4 (2)	C6—O2—C15	116.97 (19)
C8—C9—C11	117.5 (2)	C10—S1—C13	89.97 (12)
			
O1—C1—C2—C3	178.3 (2)	S1—C10—C11—C12	0.2 (3)
C6—C1—C2—C3	−0.5 (4)	Cl1—C10—C11—C9	−2.3 (4)
C1—C2—C3—C4	1.3 (4)	S1—C10—C11—C9	179.10 (19)
C2—C3—C4—C5	−0.5 (4)	O3—C9—C11—C10	−12.1 (4)
C2—C3—C4—C7	−177.2 (2)	C8—C9—C11—C10	170.1 (2)
C3—C4—C5—C6	−1.1 (4)	O3—C9—C11—C12	166.6 (3)
C7—C4—C5—C6	175.6 (2)	C8—C9—C11—C12	−11.1 (4)
C4—C5—C6—O2	−177.8 (2)	C10—C11—C12—C13	0.4 (3)
C4—C5—C6—C1	1.9 (4)	C9—C11—C12—C13	−178.6 (2)
O1—C1—C6—O2	−0.3 (3)	C11—C12—C13—Cl2	179.3 (2)
C2—C1—C6—O2	178.7 (2)	C11—C12—C13—S1	−0.8 (3)
O1—C1—C6—C5	179.9 (2)	C2—C1—O1—C14	0.1 (4)
C2—C1—C6—C5	−1.1 (3)	C6—C1—O1—C14	178.9 (2)
C3—C4—C7—C8	−175.4 (3)	C5—C6—O2—C15	1.8 (3)
C5—C4—C7—C8	8.0 (4)	C1—C6—O2—C15	−178.0 (2)
C4—C7—C8—C9	−177.4 (2)	C11—C10—S1—C13	−0.5 (2)
C7—C8—C9—O3	2.2 (4)	Cl1—C10—S1—C13	−179.29 (16)
C7—C8—C9—C11	179.9 (2)	C12—C13—S1—C10	0.7 (2)
Cl1—C10—C11—C12	178.78 (19)	Cl2—C13—S1—C10	−179.34 (18)

### Hydrogen-bond geometry (Å, °)

**Table:**

*D*—H···*A*	*D*—H	H···*A*	*D*···*A*	*D*—H···*A*
C3—H3···O3^i^	0.95	2.53	3.227 (3)	130
C12—H12···O1^ii^	0.95	2.55	3.441 (3)	157
C14—H14A···O3^iii^	0.98	2.53	3.474 (3)	161
C15—H15B···Cl1^iv^	0.98	2.82	3.647 (3)	142

Symmetry codes: (i) −*x*+1, −*y*+1, −*z*+1; (ii) −*x*+1/2, *y*+1/2, −*z*+3/2; (iii) *x*+1/2, −*y*+1/2, *z*+1/2; (iv) −*x*, −*y*+1, −*z*+1.
